# Toward a Plasmon-Based Biosensor throughout a Thermoresponsive Hydrogel

**DOI:** 10.1021/acsapm.4c02255

**Published:** 2024-11-01

**Authors:** Anne Parra, Óscar Ahumada, Andreas Thon, Valerio Pini, Julia Mingot, Elaine Armelin, Carlos Alemán, Sonia Lanzalaco

**Affiliations:** † Mecwins S.A., Ronda de Poniente, 15 2°D, Tres Cantos, 28760, Madrid, Spain; ‡ IMEM-BRT’s Group, Departament d’Enginyeria Química, EEBE, Universitat Politècnica de Catalunya, C/Eduard Maristany, 10-14, Ed. I, second floor, 08019, Barcelona, Spain; § Barcelona Research Center in Multiscale Science and Engineering, EEBE, Universitat Politècnica de Catalunya, C/Eduard Maristany, 10-14, basement S-1, 08019, Barcelona, Spain; ∥ Institute for Bioengineering of Catalonia (IBEC), The Barcelona Institute of Science and Technology, C/Baldiri Reixac 10-12, 08028, Barcelona, Spain

**Keywords:** plasmonic detection, thermoresponsive hydrogel, gold nanoparticles, biomarker classification, nanoparticle permeation, dark-field microscopy

## Abstract

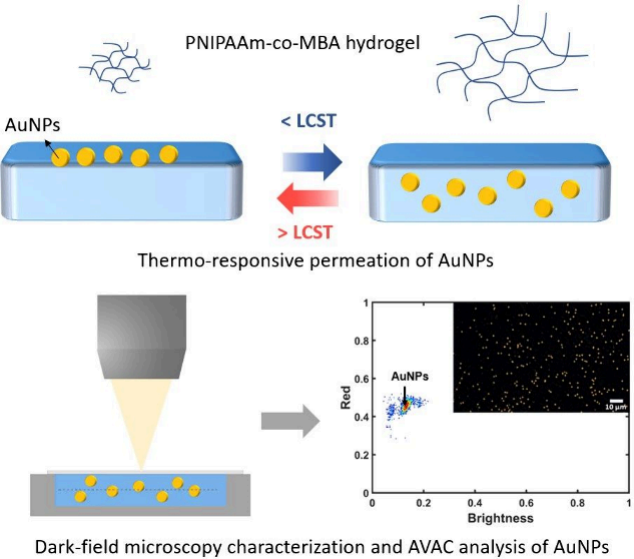

This study investigates the potential of thermoresponsive hydrogels as innovative substrates for future in vitro diagnostic (IVD) applications using AVAC technology, developed and patented by the Mecwins biomedical company. In order to convert the hydrogel in a substrate compatible with AVAC technology, the following prerequisites were established: (1) the hydrogel layer needs to be permeable to gold nanoparticles (AuNPs), and (2) the optical properties of the hydrogel should not interfere with the detection of AuNPs with AVAC technology. These two key aspects are evaluated in this work. A silicon substrate (Sil) was coated with a layer of a thermosensitive hydrogel (TSH) based on poly(*N*-isopropylacrylamide-*co*-*N*,*N*′-methylene bis(acrylamide) (PNIPAAm-*co*-MBA). The TSH offers the advantage of easy modulation of its porosity through cross-linker adjustments, crucial for the plasmonic nanoparticle (NP) permeation. The platforms, denominated as (Sil)-*g*-(PNIPAAm-*co*-MBA), were fabricated by changing the cross-linker concentrations and exploring three deposition methods: drop casting (DC), spin coating (SC), and 3D printing (3D); the DC approach resulted in a very homogeneous and thin hydrogel layer, very suitable for the final application. Furthermore, after physical-chemical characterization, the TSH demonstrated its functionality in regulating nanoparticle absorption, and AVAC technology’s capability to precisely identify such NPs through the hydrogel matrix was validated. The proposed hydrogel platform fulfills the initial requirements, opening the possibility for employing these hydrogels as dynamic substrates in sandwich immunoassay devices. The next step in the development of the hydrogel substrate would be its functionalization with biorecognition groups to allow for biomarker detection. By leveraging their enhanced capture efficiency and the ability to manipulate particle flow thermally, we anticipate a significant advancement in diagnostic methodologies, combining the spatial benefits of three-dimensional hydrogel structures with the precision of AVAC’s digital detection.

## 1 Introduction

The synergistic combination of medical and biotechnology research with engineering and science has made it possible to achieve on-target diagnostics and deep knowledge of the etiology of diseases.^1,2^ The early diagnosis and treatment of cancer and immune-deficient diseases is one of the main objectives of the scientific community and represents a major global health concern.^3^ In vitro diagnostics range from measurement/sensing technologies to next-generation sequencing (NGS), a type of DNA sequencing innovation that uses parallel sequencing of multiple small fragments of DNA to determine sequence. The first is able to detect traces of substances in bodily fluids, while the second scans the DNA of a person and its potential genomic variations.^4^ In this way, biomarker sensors hold enormous potential for early diagnosis and personalized therapy of disease.^5^ By combining modern materials or secondary label enhancement technologies, many traditional biomarker sensors based on surface plasmon resonance, SPR, and electrochemistry optimized their performance in sensitivity, limit of detection (LoD), and linear range. They could be classified into two categories: label-based and label-free sensors. The first group comprises sensors based on biomarkers decorated by secondary labels able to optimize the sensitivity by amplifying the output signals, such as fluorescent materials, magnetic beads, or even biological materials.^6^ Very recently, due to the development of materials with more complex geometries, some new secondary labels exhibited a great potential due to their better biocompatibility, larger surface-to-volume ratio, etc.^7−10^ Nano/microfabricated materials such as carbon nanotubes, graphene oxide, gold nanoparticles (AuNPs), and quantum dots (QDs) were included as secondary labels.^11−13^

AVAC technology, patented by Mecwins SA,^17^ uses plasmonic gold nanoparticles as secondary labels for the ultrasensitive detection of biomarkers. The technology is based on a sandwich immunoassay in which the secondary antibody is conjugated to AuNPs. These AuNPs are then optically detected by measuring their scattering signal with dark-field microspectrophotometry, followed by their classification and counting. The precise quantification of the AuNPs bound to the substrate surface allows the quantification of biomarkers in the range of femtograms and has been proven for different biomarkers, such as PSA, a prostate cancer recurrence biomarker, Troponin I, involved in myocardial infarction, p24, an HIV biomarker, and interleukins, involved in infectious and inflammatory diseases.

Hydrogels are gaining interest as a powerful tool in biosensing applications.^14^ Due to their open-cell microstructure and high water content, they resemble natural tissues and create an environment that stabilizes and protects biomolecules Recently, research about hydrogel-functionalized platforms enabling the detection of biomarkers is a growing trend, because they offer a noninvasive approach and a real-time solution. Among others, hydrogel microneedles integrating aptamer probes for biomarker quantification were studied by Poudineh et al., who developed a methacrylated hyaluronic acid (MeHA) biosensor based on a fluorescently labeled aptamer probe for on-needle and reagentless capture and detection of any biomarkers of interest.^15,16^ In a very appealing work, highly sensitive internal-standard surface-enhanced Raman scattering microneedles (IS-SERS-MNs) based on agarose gel were developed, to detect bacterial metabolites in interstitial fluid (ISF) as new detectable biomarkers for infection diagnosis.^17^ Convenient hydrogel patches modified with conductive materials could serve as electrochemical glucose sensors or noninvasive sweat glucose sensors for measuring natural perspiration during sedentary activities or daily living.^18,19^ Furthermore, hydrogels showed great potential in the field of on-site visual detection (colorimetric techniques).^20−23^ Shen and his team conducted a study where they developed a range of see-through poly(vinyl alcohol) (PVA) hydrogels enclosing antimony tin oxide nanoparticles (ATO NPs) and 3,3′,5,5′-tetramethylbenzidine (TMB) as the sensing material in a solid phase to identify levels of glucose and uric acid.^21^ A special type of hydrogels are responsive hydrogels, which are able to react to stimuli like pH, temperature, ionic strength, light, etc. For example, poly(*N*-isopropylacrylamide-*co*-*N*,*N*′-methylene bis(acrylamide) (PNIPAAm-*co*-MBA) is a thermosensitive hydrogel (TSH) with a lower critical solution temperature (LCST) very close to body temperature, in the range of 32–33 °C, making it attractive for a variety of biological applications.^24−26^ It has been reported to be applied for various biomedical purposes such as “on/off” switches for chemical reactions, support for cell adhesion/deadhesion, and matrices for bioseparations, among others.^27−29^ Upon heating above the LCST, the hydrogel goes from a hydrophilic to a hydrophobic state, resulting in a drastic change in the hydrogel volume caused by water expulsion, which can be tuned for the specific application.

In this work, we investigate the potential use of thermosensitive hydrogels as substrates compatible with AVAC technology, which is an innovative technology for ultrasensitive biomarker detection and quantification. More specifically, the aim is to select a suitable format of TSH that enables control of AuNP motion and allows identification of AuNPs inside the complex hydrogel matrix. To achieve this goal, the gel composition, in terms of cross-linker (MBA) concentration and polymerization time, is carefully discussed, as well as the effect of the different gel grafting methods on the structure of the hydrogel network. The final platform is an interesting starting point for further development of plasmon-based biosensors, with the next steps involving functionalization of the hydrogel with the biorecognition groups of interest for biomarker detection.

## 2 Materials and Methods

### 2.1 Materials

Silicon wafers (Sil) were supplied by Si-Mat (1 × 1 cm^2^); *N*-isopropylacrylamide (NIPAAm) monomer (purity 99%, CAS 2210-25-5), *N*,*N*′-methylenebis(acrylamide) (MBA) cross-linker (Reagent Plus 99%, CAS 110-26-9), and *N*,*N*,*N*′,*N*′-tetramethylethylenediamine (TEMED) initiator (Reagent Plus 99%, CAS110-18-9) were supplied by Sigma-Aldrich (Spain); ammonium persulfate (APS) catalyst (purity 98%, CAS 7727-54-0) was provided by Panreac S.A., and Milli-Q water grade (0.055 S cm^–1^) was used in all synthetic processes. Nitrogen gas was used for the radical polymerization reactions and was of pure grade (99.995% purity). Gold nanoparticles with a 100 nm diameter with a −COOH coating were provided by Nanopartz.

### 2.2 Plasma Treatment

For the O_2_-plasma activation of the Sil our own procedure was followed.^30^ The aim is to create new O-free radical groups on the silicon substrate by oxygen plasma treatment (PT) (plasma power 250 W, process pressure of 0.33 mbar, gas flow fixed for 180 s, and 3.2 sccm). Plasma treatment was realized with a low-pressure radio frequency (RF) plasma (80 MHz), by using a 1000 W LFG generator (Diener Electronic GmbH Co., Germany) in a 2.5 dm^3^ chamber.

### 2.3 PNIPAAm-*co*-MBA Preparation

The synthesis of the cross-linked hydrogel was carried out by preparing an initial solution containing the monomer (NIPAAm, 250 mM), the catalyst (TEMED, 2.77 mM), and the cross-linker (MBA, 10 to 100 mM). The radical copolymerization was activated by the injection of the initiator (APS, 2.77 mM), after fluxing N_2_ inside the chamber for 30 min. The procedure followed is similar to the one previously reported by the same authors.^29,31^ After different reaction times (15, 30, and 60 min) of polymerization at 30 °C, the resulting samples (indicated with the names PNIPAAm-*co*-MBA*x* (*y* min), where *x* is the millimolar concentration of MBA and *y* the minutes of polymerization time) were extracted and purified in 400 mL of Milli-Q water under stirring for 4 h, by continuous replacement of Milli-Q water, and then dried at 30 °C overnight under vacuum or by lyophilization, depending on the next characterization analyses.

### 2.4 (Sil)-*g*-(PNIPAAm-*co*-MBA) Platform Fabrication Methods

**Scheme 1:**
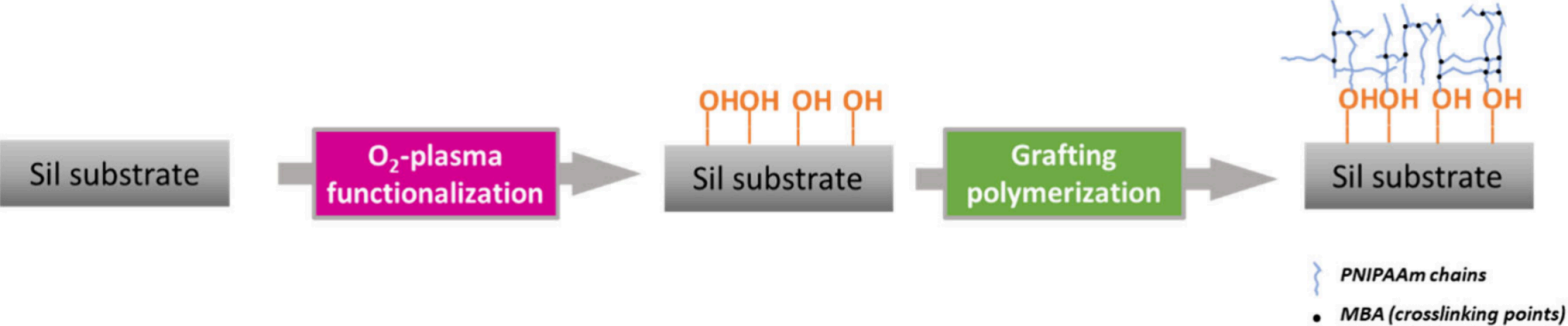
Schematic Illustration of the Main Steps of Functionalization and Grafting of the Silicon Wafers

The deposition of the PNIPAAm-*co*-MBA hydrogel onto the Sil was performed by taking advantage of the oxidative activity and of the etching introduced on the substrate surface by the O_2_-plasma treatment. Scheme 1 illustrates the main steps of functionalization and grafting realized on the silicon wafer. The general mechanism is similar to that previously reported for the PNIPAAm-*co*-MBA grafting onto the polymer support.^30,31^ Briefly, the functionalization of the silicon surface by the O_2_-plasma is responsible for the formation of oxidative functional groups (i.e., COOH, OH, ...) that initiated the graft copolymerization of the NIPAAm monomer.

The drop casting (DC) was carried out by depositing 0.3 mL of the copolymer solution, described in Section 2.3, onto the wafer previously plasma treated and laid down in a mold inserted in a flask under a controlled atmosphere (N_2_ environment).

The spin coating (SC) has been carried out using a spin-coater model ws-400bz-6npp from Laurell Technologies Corporation. The Sil wafer was fixed onto the spin coater plate under a vacuum. A fixed volume (20 μL) of the PNIPAAm-*co*-MBA10 hydrogel, previously prepared following the protocol described in Section 2.3, was deposited onto the wafer, and three following cycles at 800 rpm for 3 min were applied to achieve the final homogeneous deposition.

Three-dimensional printing by fused deposition modeling (FDM), among which stands out the printing of thermosensitive platforms, also known as 4D printing, has been employed for the hydrogel deposition onto the Sil wafer. An Axo A1 printer was used, based on pressure extrusion for hydrogels and bioinks, with a resolution of 1.25 μm per layer or position, and extrusion conditions can be controlled with high-temperature (up to 250 °C) or low temperature (from 8 to 60 °C) heads, in addition to a head for cross-linking or curing with ultraviolet light at 365, 395, or 405 nm. Herein, the PNIPAAM-*co*-MBA10 hydrogel, previously prepared following the protocol described in Section 2.3, was printed at room temperature on the Sil surface using a 21G blunt needle with a printhead air pressure of 2 psi, following an aligned rectilinear pattern until the surface was fully covered.

### 2.5 Physical-Chemical Characterization

Water contact angle (WCA) measurements were conducted using the sessile water drop method at room temperature and controlled humidity in order to establish the hydrophilicity of the surface. Measurements were carried out with the equipment of the OCA 20 (DataPhysics Instruments GmbH, Filderstadt), and the software SCA20 was used to analyze the data acquired (*n* > 10).

UV–vis measurements of the dispersion of AuNPs were taken using a Cary 100 Bio UV–visible spectrophotometer from Agilent Technologies (Santa Clara, CA, USA). The spectral range from 400 to 800 nm was investigated and measured in one-nanometer steps.

Fourier transform infrared (FTIR) spectra were recorded on an FTIR Jasco 4100 spectrophotometer. Samples were deposited on an attenuated total reflection accessory (top plate) with a diamond crystal (Specac model MKII Golden Gate Heated Single Reflection Diamond ATR). For each sample, 64 scans were performed between 4000 and 600 cm^–1^ with a resolution of 4 cm^–1^.

Raman spectra were acquired using a Renishaw dispersive Raman microscope spectrometer (model In Via Qontor, Germany) and Renishaw WiRE software. The spectrometer is equipped with a Leica DM 2700 M optical microscope, a thermoelectrically cooled charge-coupled device (CCD) detector (−70 °C, 1024 × 256 pixels), and a scattered light spectrograph with a 2400 lines/mm grating. The experiments were performed with a 532 nm excitation wavelength and with a nominal laser output power of 300 mW. The exposure time was 10 s, the laser power was adjusted to 1% of its nominal output power, and each spectrum was collected with 3 accumulations. All Raman spectra were collected in a spectral range from 400 to 4000 cm^–1^ with the same measurement parameters.

Scanning electron microscopy (SEM) imaging was conducted on lyophilized samples using a focused ion beam Zeiss Neon 40 scanning electron microscope equipped with an energy-dispersive X-ray analysis (EDX) spectroscopy system operating at 5 kV. Samples were sputter-coated with a thin layer of carbon to prevent sample charging problems. The diameter of the pores of the hydrogels was measured with ImageJ software (*n* = 100).

X-ray photoelectron spectroscopy (XPS) analyses were used for the detection of chemical species of PNIPAAm-*co*-MBA samples grafted onto the wafers. The assays were performed on a SPECS system equipped with an Al anode XR50 source operating at 150 mW and a Phoibos MCD-9 detector. The pressure in the analysis chamber was always below 10–7 Pa. The pass energy of the hemispherical analyzer was set at 25 eV, and the energy step was set at 0.1 eV. Data processing was performed with the Casa XPS program (Casa Software Ltd., UK).

### 2.6 Dark Field Imaging of AuNPs in TSH

Grafted TSH samples were incubated with a solution of 5 μg/mL AuNPs for at least 1 h. To homogenize the hydrogel layer and control its thickness, spacers and a microscope coverslip were placed next to and on top of the hydrogel, respectively. Dark field microscopy was used for AuNP detection (Eclipse Ni microscope, Nikon) with a 20×/0.45A objective and 20 ms exposure time, and images were taken with a scientific-grade CMOS RGB camera (Ximea MC124CG-SY-UB). The raw images were corrected by subtracting the scattering originating from the substrate and analyzed with AVAC technology.

## 3 Results and Discussion

### 3.1 Overview of the Plasmon-Based Platform Assembly

**Scheme 2:**
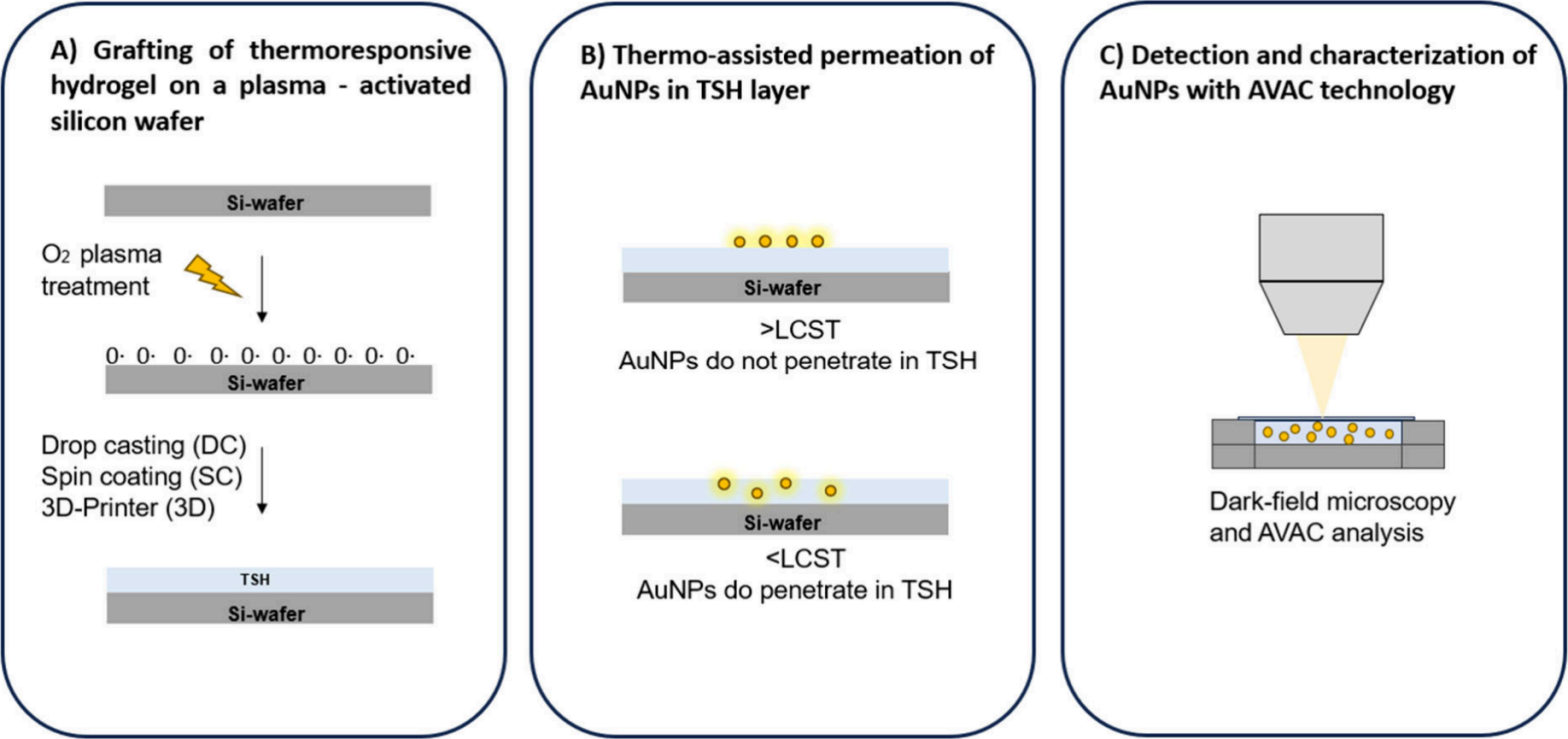
Schematic Illustration of (A) the Pathways Followed for the (Sil)-g-(PNIPAAm-co-MBA*x*)/*y* Platform Preparation; (B) the Thermo-Assisted AuNP Permeation Experiments Carried out with Different TSHs; and (C) the Detection and Characterization of AuNPs inside the TSH, Using Scattering with Dark-Field Microspectrophotometry

Scheme 2 summarizes the experimental work carried out in this study, which consists of three main steps: (A) preparation of TSH on the Sil substrate, previously treated by an O_2_-plasma to both clean and functionalize the surface, by means of different technologies (DC, SC, and 3D). The final platform will be indicated with the acronym (Sil)-*g*-(PNIPAAm-*co*-MBA*x*)/*y*, where *x* is the amount of cross-linker employed (in mM) and *y* is the technology employed to deposit the TSH. Therefore, in the sequence of Scheme 2, (B) represents the evaluation of the thermo-assisted permeation of the (Sil)-*g*-(PNIPAAm-*co*-MBAx)/y platforms, changing the porosity of the TSH;, and (C) illustrates the in situ detection and characterization of plasmonic NPs immobilized inside the PNIPAAm-*co*-MBA hydrogel, by means of dark-field microspectrophotometry.

### 3.2 Silicon Wafer Treated by Low-Pressure O_2_-Plasma

**Figure 1:**
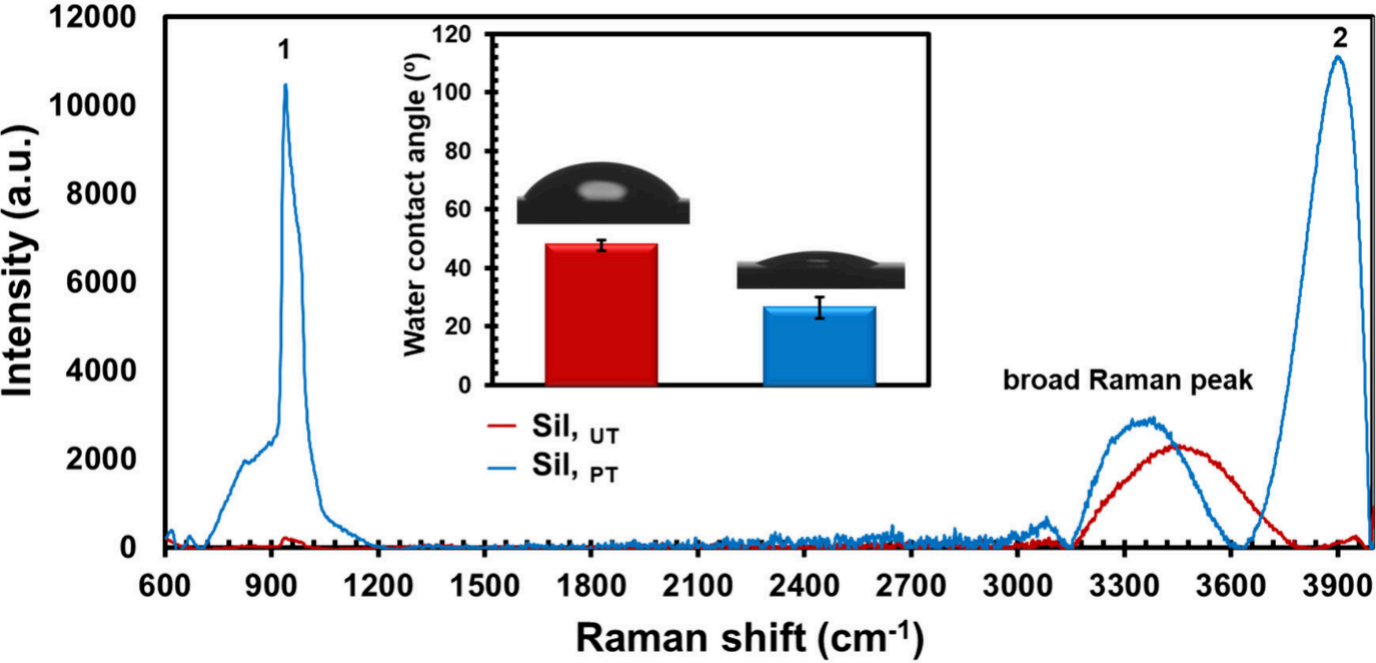
Raman spectra and, in the inset, water contact angle (WCA) measurements carried out on untreated (Sil_UT_) and plasma-treated (Sil_PT_) silicon wafers.

In order to covalently attach the TSH onto the silicon wafer, an initial plasma treatment in the presence of oxygen gas was carried out to promote the formation of oxidative chemical groups suitable for the following grafting reaction. In addition to chemical functionalization, plasma causes etching that leads to physical changes of the wafer surface by means of a combination of both physical and chemical reactions.^32,33^ Raman spectroscopy is highly efficient for plasma etching analysis due to the fact that the scattering process involves electron–phonon interactions, which could provide highly relevant information.^17,34−36^ However, Raman analysis of plasma-treated silicon is poorly presented in the literature. The operative conditions of the plasma treatment carried out in the present work are reported in the Materials and Methods section. Figure 1 shows the Raman spectra for the untreated wafer, Sil_UT_, and the plasma treated Sil, Sil_PT_.

Easily noticeable is the increase of the intensity of the peaks located at ∼950 cm^–1^ (1) (its second harmonic at 521 cm^–1^ is reported in Figure S1) and at 3900 cm^–1^ (2), which originate from the silicon substrate. The increase in intensity of such absorption bands is due to the etching process, which makes the surface of the first layer of the wafer more accessible. In addition, a broad Raman peak located at ∼3450 cm^–1^, observed for Sil_UT_, appears and is attributed to electronic Raman scattering shifts to the low-energy side of the spectra, as the plasma treatment is carried out, Sil_PT_.^33^ In general, the main process in electronic Raman scattering has single-particle and collective plasmon excitation.^37^ Keeping in mind that the silicon-based material employed herein presents insulating properties, we assumed that no plasmons are present. The evident changes observed in the Raman spectra highlight the etching efficiency.

In order to investigate chemical modification of the surface, water contact angle (WCA) measurements of Sil_UT_ and Sil_PT_ were carried out and are reported in the inset of Figure 1. In agreement with the existing literature,^38^ Sil_UT_ exhibits a good hydrophilicity before the treatment (with a WCA of 48 ± 2°). Samples treated with low-pressure O_2_-plasma experience a significant increase of wettability to a WCA of 26 ± 3°, indicating a surface functionalization of the wafer and the formation of new oxidative functional groups suitable for the next step of TSH grafting.

### 3.3 Fabrication and Characterization of Sil-*g*-(PNIPAAm-*co*-MBA) Platforms

**Figure 2:**
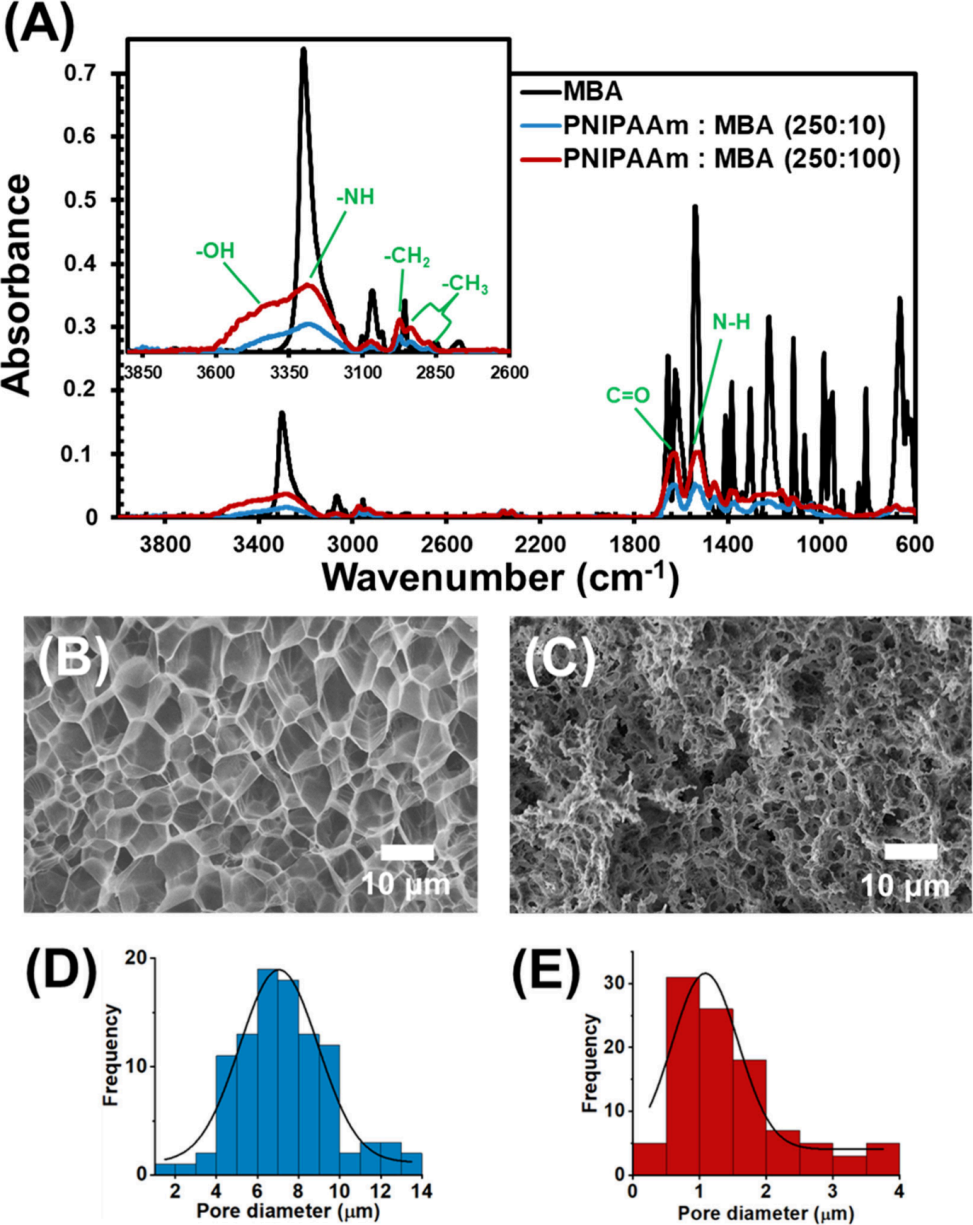
(A) FTIR spectra showing the comparison between bands associated with the cross-linker (MBA) and the PNIPAAm copolymers obtained in the presence of different concentrations of MBA, PNIPAAm-*co*-MBA10 with NIPAAm:MBA = 250:10, and PNIPAAm-*co*-MBA100 with NIPAAm:MBA = 250:100; SEM micrographs and pore size distribution of (B, D) PNIPAAm-*co*-MBA10 and (C, E) PNIPAAm-*co*-MBA100.

Before studying the graft reaction of PNIPAAm-*co*-MBA onto the wafers, the best TSH for the specific application has been selected, by studying the influence of the cross-linker (MBA) concentration on the polymerization of the hydrogel. A series of reactions were carried out at 30 °C for different times (15, 30, and 60 min), using a NIPAAm monomer concentration of 250 mM, APS/TEMED = 1:1 ([APS] = 2.77 mM), and cross-linker concentration of 100 mM. The high ratio between the monomer and the cross-linker (250:100) was selected in order to reduce the porosity of the hydrogel and therefore limiting the AuNPs’ passage after the collapse of the hydrogel. As reported in Figure S2, the normalized FT-IR spectra showed several changes between 2800 and 3800 cm^–1^, as the reaction time increases. More in detail, the bands observed in the range 2800–3000 cm^–1^, attributed to the stretching vibration of −CH_2_ and −CH_3_ groups, enhance with the reaction time, while those between 3200 and 3600 cm^–1^, related to −OH groups and to the stretching vibration of the −NH, diminish.^31^ These results suggest that samples obtained at the lowest time investigated (15 min) are more hydrophilic due to the presence of a low amount of isopropyl groups coming from the monomer, that is, due to a high amount of cross-linker. These observations are in good agreement with literature, where it is frequently reported that the reaction rate of the cross-linker (MBA) is higher than that of the monomer (NIPAAm), justifying its faster and larger addition during the first minutes of the polymerization, if compared with the monomer addition.^39^ In order to improve the amount of NIPAAm molecules converted at low time, the amount of MBA was decreased to 10 mM, with a final ratio NIPAAm:MBA of 250:10, maintaining a reaction time of 15 min. FT-IR analyses reported in Figure 2A show a comparison between the NIPAAm monomer and the copolymers PNIPAAm-*co*-MBA10 and PNIPAAm-*co*-MBA100, where 10 and 100 are the concentrations of MBA in mM used. Both samples presented characteristic peaks of the copolymer, whereas no trace of unreacted monomer has been observed, even with the short reaction time employed. More in detail, PNIPAAm-*co*-MBA10 and PNIPAAm-*co*-MBA100 present the absorption bands at 1512 and 1620 cm^–1^, assigned for the N–H of amide and C=O stretching of amide groups, respectively, as well as the absorption peaks between 2850 and 3000 cm^–1^ (−CH_3_ and −CH_2_– groups), and the broad bands in the range between 3290 and 3500 cm^–1^ attributed to −OH and −NH vibrations, respectively.^31^ By comparing the ratio of the absorbance of −CH_3_ and −NH_2_ bands, we observed that this value decreases from 0.45 to 0.35, from PNIPAAm-*co*-MBA10 to PNIPAAm-*co*-MBA100; the higher the cross-linker concentration, the greater the amount of −NH functionalities in the final hydrogel.

The morphology and pore size of the hydrogel network, depending on the cross-linker concentration, are reported in Figure 2B–E. The images of the cross-section of lyophilized samples show that a uniform and porous structure with large pores is obtained for PNIPAAm-*co*-MBA10 (Figure2B), while a dense structure with very small pores was achieved for PNIPAAm-*co*-MBA100 (Figure 2C). Thus, the average pore size, which was determined using several random locations from three micrographs, was 7.3 ± 2.3 and 1.9 ± 0.8 μm for PNIPAAm-*co*-MBA10 (Figure 2D) and for PNIPAAm-*co*-MBA100 (Figure 2E), respectively. Moreover, the PNIPAAm-*co*-MBA hydrogels present a well-interconnected pore network, which is beneficial for water transfer and, consequently, for the thermoresponsive behavior of the (Sil)-*g*-(PNIPAAm-*co*-MBA) platforms.

**Figure 3:**
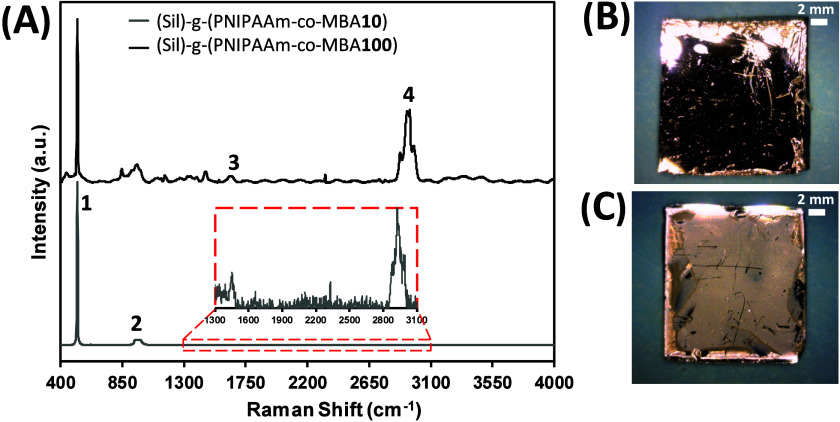
(A) Raman spectra and corresponding optical images of (B) (Sil)-*g*-PNIPAAm-*co*-MBA10 and (C) (Sil)-*g*-PNIPAAm-*co*-MBA100.

The plasma treatment proved to be crucial for the adhesion of PNIPAAm-*co*-MBA to the silicon wafer. Wafers without oxygen-plasma treatment were not able to maintain the gel adhered to their surfaces due to the lack of polar anchoring groups, as previously reported.^30^ Therefore, after gel deposition and purification processes, the PNIPAAm layer was easily removed from the untreated substrate, while it was perfectly adhered when the plasma treatment was carried out. In order to corroborate the presence of the covalently grafted hydrogel, the growth of PNIPAAm-*co*-MBA molecules was monitored by Raman at a low polymerization time (15 min), after plasma treatment. Figure 3A shows the Raman spectra of (Sil)-*g*-(PNIPAAm-*co*-MBA10) and (Sil)-*g*-(PNIPAAm-*co*-MBA100). Peaks at 571 cm^–1^ (1) and 950 cm^–1^ (2) originating from the silicon substrate are clearly observed for both samples, while peaks related to the thermosensitive hydrogel, which are centered at 1460 (3) and 2930 cm^–1^ (4) and corresponding to the C–H bending and CH_3_ stretching, respectively,^40−42^ are more easily identified in (Sil)-*g*-(PNIPAAm-*co*-MBA10) than in (Sil)-*g*-(PNIPAAm-*co*-MBA100). It is necessary to enlarge the spectrum to be able to observe the hydrogel peaks in (Sil)-*g*-(PNIPAAm-*co*-MBA100) due to the thinner layer obtained with the highest MBA concentration. At the low polymerization time employed, the entire surface of the wafer is covered by the hydrogel (Figure 3B,C) with different thicknesses, 75 ± 2.5 and 32 ± 1.8 μm for (Sil)-*g*-(PNIPAAm-*co*-MBA10) and (Sil)-*g*-(PNIPAAm-*co*-MBA100), respectively.

For more evidence of the surface composition, XPS was employed to confirm the grafting of the hydrogel onto the wafer, showing that the N and O concentration increases at the highest amount of MBA investigated. Typical spectra and atomic concentration of PNIPAAm copolymerized with MBA were observed, and a detailed discussion is reported in the Supporting Information (Figure S3 and Table S1).

### 3.4 Thermo-controlled Permeation of AuNPs in TSH

The selection of the TSH comes from its ability to regulate the penetration of the AuNPs inside its structure by thermo-controlled opening/closing of the gel pores. There are several examples of PNIPAAm polymer grafted with metallic NPs, nanorods, nanofibers, and other hybrid materials, in which the proved thermoregulation capability offered by this TSH was reported.^43−45^ However, scarce works have investigated the good permeability of PNIPAAm, as host soft material, toward Au NP diffusion. For instance, Chang et al. prepared a biosensor array anchoring the TSH to the substrate and, then, AuNPs were introduced using a “breathing-in” process of swelling and shrinking by modulating the temperature of both the PNIPAAm-based array and that of the AuNP solution.^46^ Further incorporation of biotin molecules allowed the system to act as a label-free biosensor to quantify streptavidin protein by means of near-IR wavelength shifts. Comparing the changes in the localized surface plasmon resonance (LSPR) peak position, given by the metal-NPs, and the interaction with the target molecule, they demonstrated the diffusion of AuNPs induced by thermal mobility. Contrarily to previous studies, AVAC technology does not need any target molecule for the plasmon resonance effect detection, and the quantification of biomarkers is promoted by color variations in optical microscopes, as will be exemplified in Section 3.6.

**Figure 4:**
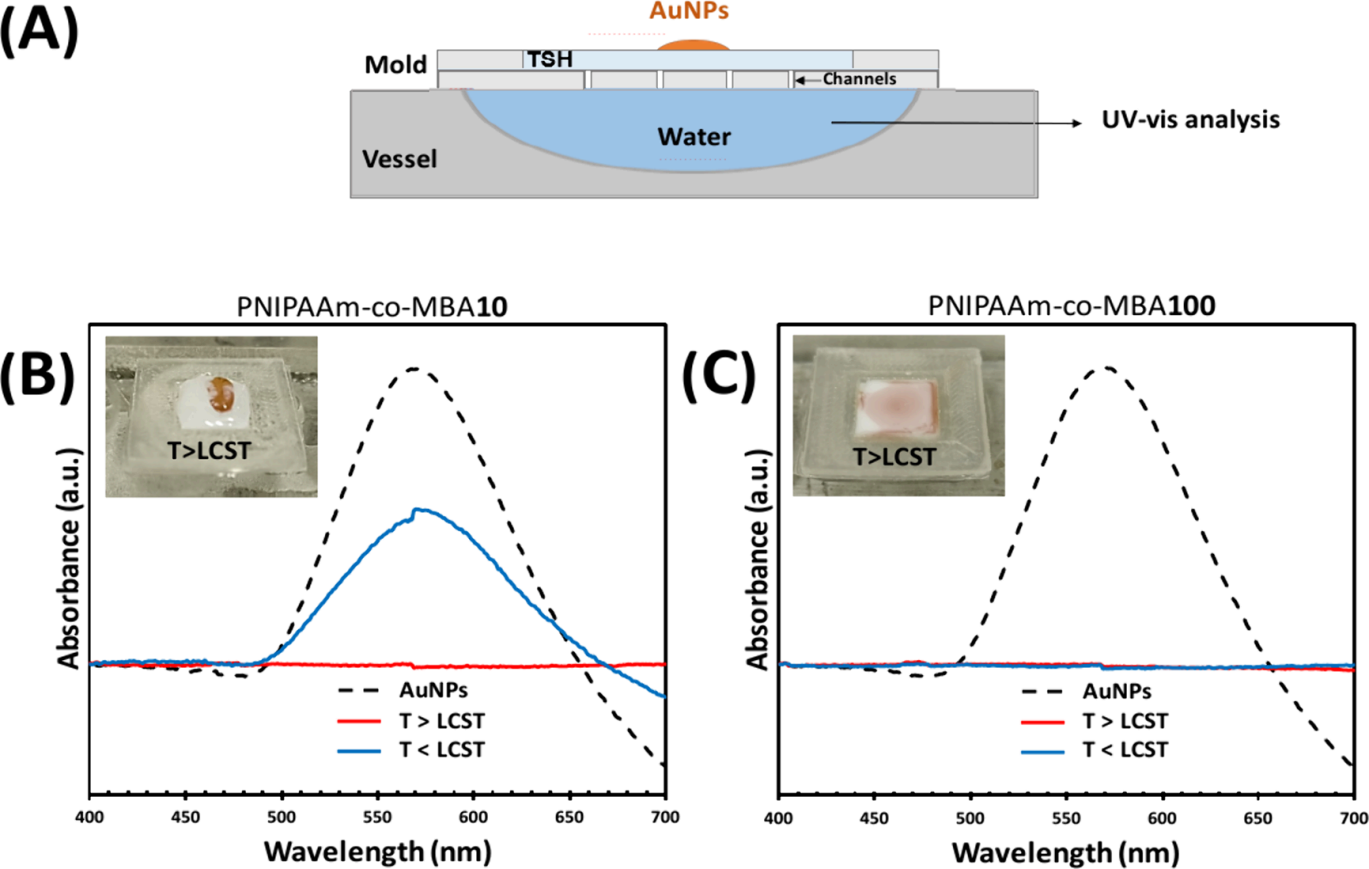
(A) Illustration of the experimental setup for the thermo-assisted AuNP permeation assays; (B, C) UV–vis spectra of (Sil)-*g*-(PNIPAAm-*co*-MBA10) and (Sil)-*g*-(PNIPAAm-*co*-MBA100) platforms, respectively. Insets in parts (B) and (C) correspond to optical images of the AuNP solution distribution at *T* > LCST above PNIPAAm-*co*-MBA10 and PNIPAAm-*co*-MBA100 hydrogels, respectively.

Previously, the thermocontrolled permeation of AuNPs in the PNIPAAm hydrogel was followed with UV–visible absorbance responses. Figure 4A reports the results of the thermo-assisted permeation assays carried out using PNIPAAm-*co*-MBA10 and PNIPAAm-*co*-MBA100 hydrogels. The aim of such assays is to investigate the distribution of the AuNPs inside the porous structure of the TSH, studying its dependence on the internal porosity and on the temperature applied. A fixed amount of AuNP solution (20 μL) is deposited onto a layer of TSH that is laid down in a mold and put in contact with a vessel containing pure water (Figure 4A), maintained at 40 °C (*T* > LCST). The water is then analyzed by UV–vis spectrophotometry to understand whether the AuNPs crossed through the TSH. A different distribution of the droplets deposited onto the surface of the TSH has been observed. Insets of Figure 4B,C report optical images of both TSHs at 40 °C, showing that the drop maintains its shape on the hydrophobic surface of the PNIPAAm-*co*-MBA10, while it spreads in the case of PNIPAAm-*co*-MBA100, suggesting, in the last case, a more hydrophilic surface that better interacts with the aqueous AuNP solution. It agrees with the results observed by spectroscopic investigation (Figure 2A), which revealed a higher amount of −NH groups in the PNIPAAm-*co*-MBA100 sample, responsible for conferring higher hydrophilicity to the final copolymer. To confirm this, water contact angle tests at *T* > LCST were performed and are reported in Supporting Information (Figure S4), confirming that the higher amount of cross-linker increased the wettability of the hydrogel. The value of the contact angle decreased from 76.3 ± 4.2 to 45.2 ± 2.6 from PNIPAAm-*co*-MBA10 to PNIPAAm-*co*-MBA100.

Keeping the temperature constant at 40 °C, no motion of the AuNPs inside both hydrogels was observed, and no peak of AuNPs was detected by UV–vis spectroscopy (red line, Figure 4B,C), if compared with the absorbance of the AuNP solution (115 μg/mL AuNPs) located at ∼570 nm (black line, Figure 4B,C). The shrinkage of the porous structure at *T* > LCST was enough to prevent the passage of the nanoparticles for both copolymers investigated. Progressively, the temperature was decreased until 25 °C (*T* < LCST) to help the solution to penetrate inside the porous structure of the TSH. After 10 min, UV–vis curves revealed the appearance of the AuNPs’ peak, corresponding to a AuNP concentration of 58 μg/mL, in the solution withdrawn from the experiment carried out with PNIPAAm-*co*-MBA10 gel, whereas in the case of PNIPAAm-*co*-MBA100 such absorption was not detected (blue line, Figure 4B,C), confirming that the larger pores obtained at the lowest cross-linker concentration facilitate the crossing of the nanoparticles through the gel matrix. On the other hand, the denser structure obtained with PNIPAAm-*co*-MBA100 hinders the AuNPs from passing through at high temperatures due to the TSH shrinkage, and the NPs were trapped on the top of the TSH. Von Klitzing and co-workers reported many years ago the influence of counterions of Au nanomaterial in PNIPAAm brushes.^46^ They found different plasmon color shifts in the UV–vis spectrum upon increasing the temperature from 20 to 45 °C. By employing neutron reflectivity (NR) measurements in D_2_O, they demonstrated Au core–shell NPs stabilized with citrate anions are well dispersed with the PNIPAAm brushes at 20 °C. In contrast to PNIPAAm/AuNP-citrate, the reflectivity curve of swollen PNIPAAm/AuNP-MDA (12-mercaptododecanoic acid), i.e., AuNPs stabilized with a more hydrophobic shell, shows the NPs remain at the brush surface. Interestingly, in the collapsed state (*T* > LCST), both systems presented similar reflectivity curves and scattering length density profiles. We cannot compare our gel macroscopic thicknesses with the PNIPAAm brushes (100 nm in the expanded structure) cited above. However, we can affirm our Au-NPs do not present any change in the swollen (below LCST) or collapsed states (above LCST) in the UV–vis spectra, which confirms the solution penetration is mainly governed by the intermolecular forces of PNIPAAm chains with water. Prompted by these results, the PNIPAAm-*co*-MBA10 gel has been selected as the most appropriate composition for the plasmon-based device, since it is able to ensure a thermo-controlled motion of the AuNPs across the TSH structure.

### 3.5 Looking for the Best Strategy to Graft the TSH onto the Silicon Wafer

Many efforts are devoted to the development of customized hydrogel-based devices, as layered and blended organic–inorganic cocktails designed to provide an optimal sensing environment, like that reported by Kim et al.^42^ Yet, the importance of the deposition method to bring such layers to their full functionality is too often underestimated. In fact, many times, critical sensor performance relies on the special features obtained through the deposition method chosen. Furthermore, this dependence on a deposition or fabrication method may become the sole reason that lab-scale sensors will not be easily adapted to large-scale production because their performance will inherently decrease. Therefore, herein we also present a preliminary screening of three production methods, two conventional, as drop-casting and spin-coating, and one innovative, as 3D-printing, very suitable for large-scale application. In all cases, Sil-*g*-(PNIPAAm-*co*-MBA10) platforms have been prepared once the best TSH had been selected as described in the previous section. The operative conditions are explained in the Materials and Methods section.

**Figure 5:**
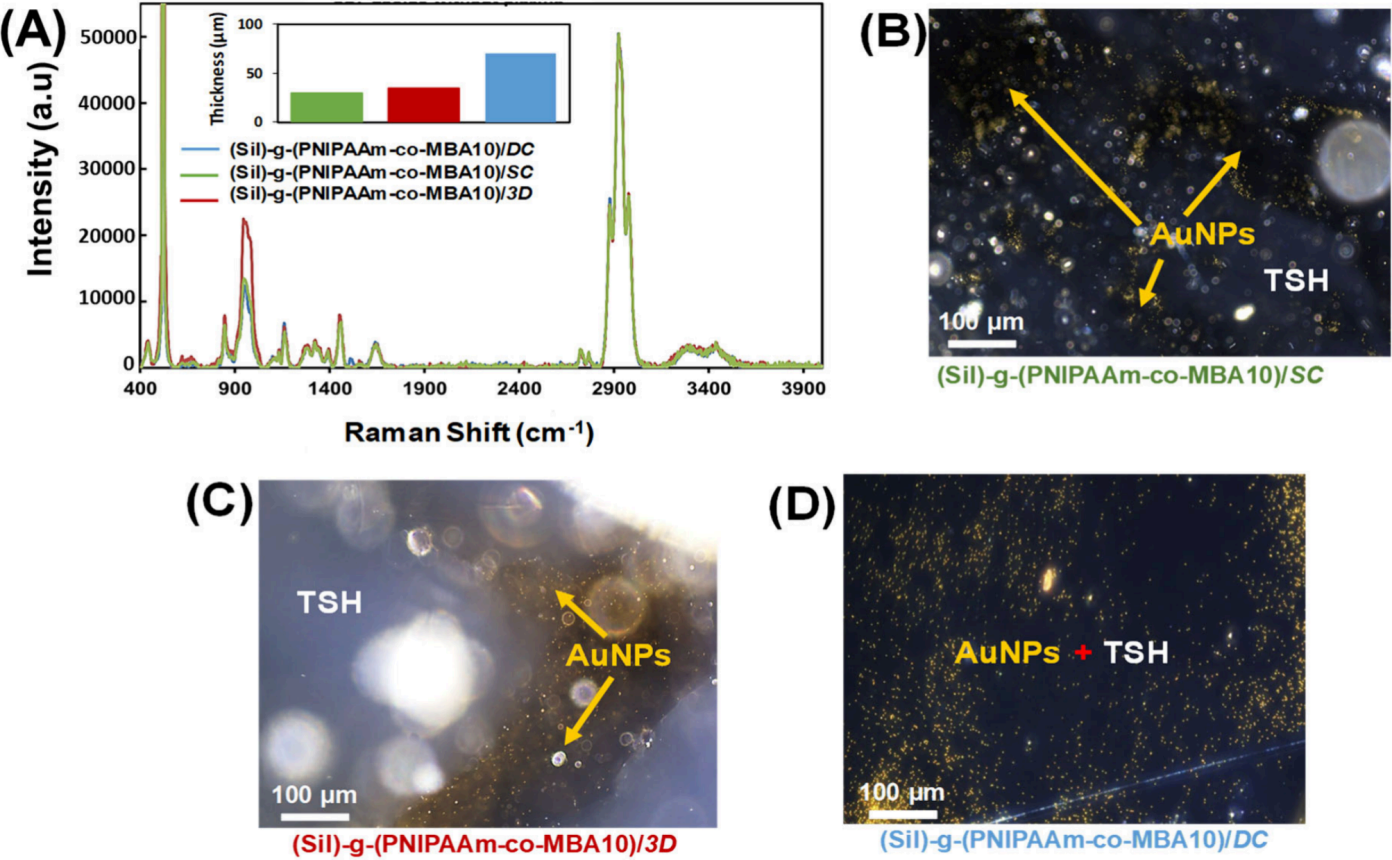
(A) Raman spectra showing the comparison between (Sil)-*g*-(PNIPAAm-*co*-MBA10)/DC, (Sil)-*g*-(PNIPAAm-*co*-MBA10)/SC, and (Sil)-*g*-(PNIPAAm-*co*-MBA10)/3D platforms (inset: thickness of the three plasmon-based devices). Dark-field microcopy images of (B) (Sil)-*g*-(PNIPAAm-*co*-MBA10)/SC, (C) (Sil)-*g*-(PNIPAAm-*co*-MBA10)/3D, and (D) (Sil)-*g*-(PNIPAAm-*co*-MBA10)/DC, showing the AuNPs distribution.

Briefly, the (Sil)-*g*-(PNIPAAm-*co*-MBA10)/DC is prepared in a one-step methodology, by radical polymerization in situ of the hydrogel using an inert environment (under N_2_), while (Sil)-*g*-(PNIPAAm-*co*-MBA10)/SC and Sil-*g*-(PNIPAAm-*co*-MBA10)/3D are obtained by a two-step technique, consisting in a previous polymerization of the TSH, later deposited onto the silicon wafer, before the complete gelation. Figure 5A reports the Raman spectra of the three devices obtained and shows that the typical peaks of the TSH, as shown in Figure 3, are identified for all samples. The hydrogel layer was successfully deposited on the silicon substrate. However, in the case of (Sil)-*g*-(PNIPAAm-*co*-MBA10)/3D the peak at 950 cm^–1^, attributed to the silicon substrate, is more pronounced compared to (Sil)-*g*-(PNIPAAm-*co*-MBA10)/DC and (Sil)-*g*-(PNIPAAm-*co*-MBA10)/SC. This observation suggests that the distribution of the TSH is not completely uniform for the 3D deposited sample, the support surface being more exposed to the Raman laser. The thickness of the three TSH layers has been calculated, being 30 ± 1.6 μm, 35 ± 1.6 μm, and 75 ± 2.5 μm for (Sil)-*g*-(PNIPAAm-*co*-MBA10)/SC, (Sil)-*g*-(PNIPAAm-*co*-MBA10)/3D, and (Sil)-*g*-(PNIPAAm-*co*-MBA10)/DC, respectively. To better investigate the compatibility of TSH and the AuNPs in terms of particle distribution, optical images obtained by dark-field micrographs of swollen hydrogels containing AuNPs were taken and are reported in Figure 5B–D. As it is observed, the AuNPs are uniformly distributed when (Sil)-*g*-(PNIPAAm-*co*-MBA10)/DC is employed (Figure 5D), while for (Sil)-*g*-(PNIPAAm-*co*-MBA10)/SC (Figure 5B) and (Sil)-*g*-(PNIPAAm-*co*-MBA10)/3D (Figure 5C) agglomerates and holes are observed, respectively. The two-step methodologies do not ensure a uniform distribution of the TSH, mainly in the case of the 3D printed technique; the presence of big wells where particles prefer to accumulate is observed. For the current investigation, the one-step methodology (DC) is identified as the best coating strategy.

### 3.6 Detection of AuNPs in the Hydrogel Matrix

**Figure 6:**
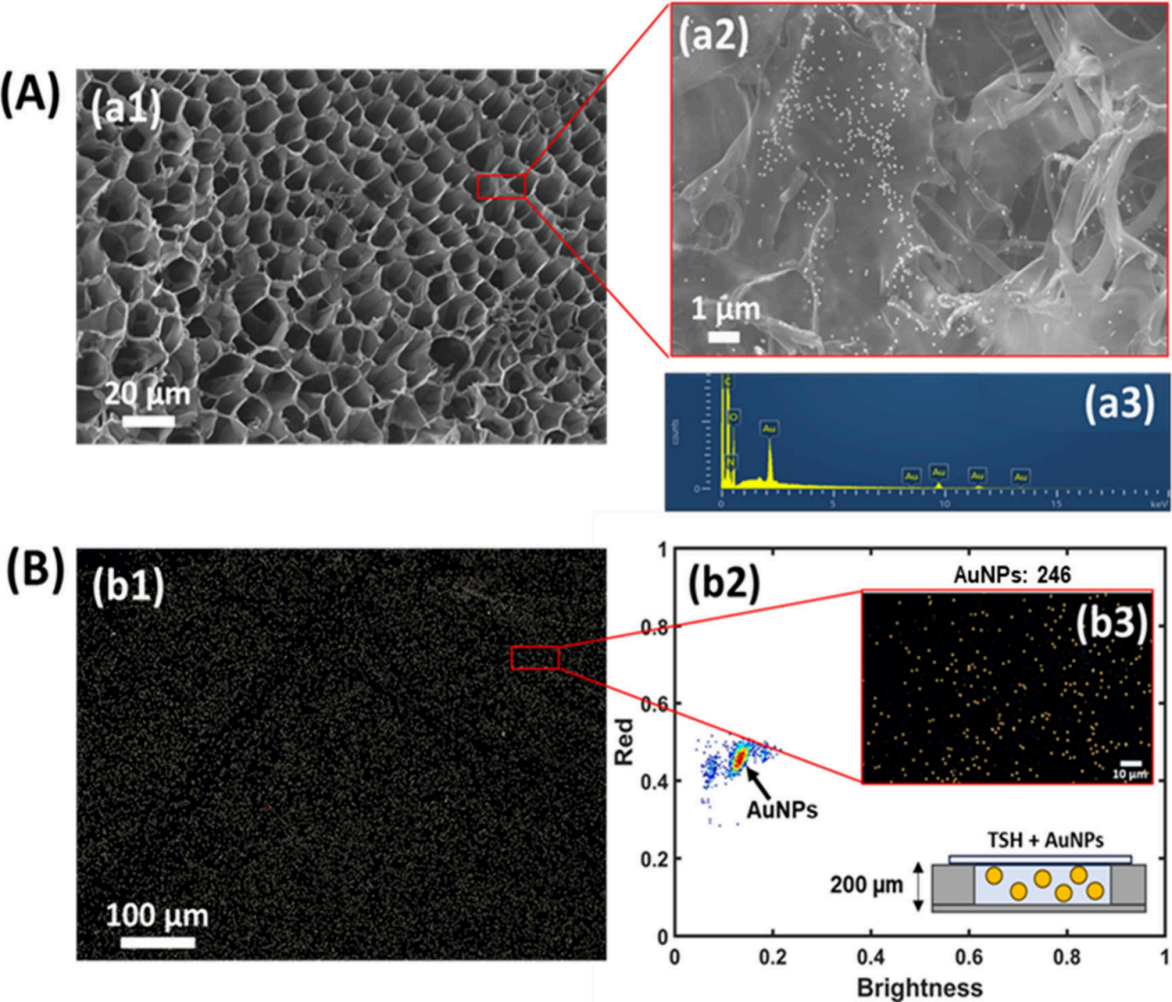
AuNP identification by means of (A) SEM micrograph and EDX spectrum and (B) dark-field micrograph using AVAC technology and color 2D-histogram. Cryogenic SEM micrographs of (Sil)-g(PNIPAAm-*co*-MBA10)/DC at (a1) 500× and (a2) 6500× after AuNP capture; (a3) EDX elemental analysis of the (Sil)-g(PNIPAAm-*co*-MBA10)/DC sample; (b1) dark-field image and (b2) two-dimensional histogram corresponding to AuNPs embedded in the hydrogel layer; (b3) dark-field microscopy image corresponding to the two-dimensional histogram, where 246 AuNPs have been detected. The bottom inset is a schematic drawing of the experimental setup.

The optical detection of plasmonic AuNPs, which are used as secondary antibody labels, is one of the key elements of AVAC technology and confers its ultrahigh sensitivity. Consequently, it is crucial to evaluate the effect of the hydrogel layer on the detection of AuNPs. For this purpose, dark-field microscopy images of AuNPs in the hydrogel layer were analyzed with AVAC technology. As the described hydrogel does not contain biorecognition groups, nonfunctionalized AuNPs were used for this study. The distribution of the AuNPs around the intricate internal structure of the hydrogel was also investigated. Cryogenic scanning electron microscopy images of the hydrogel (Figure 6a1), after AuNP incubation, demonstrated a homogeneous distribution around the intricate and shaped structure of the pores (Figure 6a2), perfectly adhered to the pore walls. EDX analysis (Figure 6a3) confirmed the presence of Au, besides elements attributed to the hydrogel (C, O, and N).

Quite interestingly, by means of AVAC technology, similar images were obtained by using their scattering signal with dark-field microspectrophotometry, which perfectly allows the identification of the plasmonic nanoparticles. AVAC technology allows us to characterize AuNPs in dark-field microscopy images based on their specific scattering signal. When analyzing a dark-field microscopy image with AVAC technology, a two-dimensional histogram is obtained, where the brightness and a color component of each found particle are represented. Figure 6b1–b3 show the dark-field image and the corresponding two-dimensional histogram (Figure 6b2) of AuNPs embedded in a (Sil)-g(PNIPAAm-*co*-MBA10)/DC sample. Particle brightness (scattering) is represented on the horizontal axis and the red color component on the vertical axis, both normalized. The number of particles with a certain combination of brightness and color is given using a color-coded scale, from none (white) or few (blue, green), to many (yellow, red).

The AuNP population is clearly visible on the two-dimensional histogram, indicated with an arrow, confirming that the AuNPs embedded in the hydrogel network can be detected with AVAC technology. This result shows that the optical properties of the hydrogel are compatible with in situ AuNP detection and pave the way for the application of this simple and versatile technology in future applications of quantitative plasmonic biosensors based on a hydrogel.

Finally, Table 1 reports an overview of previous studies that proposed the use of a PNIPAAm-based hydrogel to construct a biosensor. Within applications of PNIPAAm hydrogels to biosensors, different functions of the polymer have been developed. The most commonly explored areas are drug delivery (49% of articles) and tissue engineering (29%), while applications in biosensing are described less frequently (5% of research articles).^26^ To the best of our knowledge, no previous studies have proposed the use of a PNIPAAm-based hydrogel as a biosensor for biomarkers, where detection is performed by optical detection of plasmonic gold nanoparticles, as in the present work.

**Table 1:** Comparison of Biosensors for Biomarkers Based on PNIPAAM as a TSH

**TSH function**	**TSH composition**	**Detection method**	**Ref**
Thermoreversible adsorption of antibodies in the surface that allows to interchange and renew the sensing interface	PNIPAAm	Electrochemical	(47)
Ability to bind antigens reversibly, allowing for the reuse of these antibodies	PNIPAAm brushes	Quartz crystal microbalance, fluorescence, electrochemical impedance	(48)
Photopatterned hydrogels with high resistance to protein and cell adhesion, for immunoassays in whole blood	PNIPAAm-*co*-PAA	Fluorescence	(49)
Conjugation to the antibody–antigen complex thermally precipitated, resulting in biomarker enrichment	PNIPAAm-*co*-*N*-(2-hydroxyisopropyl) acrylamide-*co*-strained alkyne-isopropylacrylamide	Lateral flow immunoassay	(50)
Sensor surface modification by adsorption of preformed microgel–enzyme complexes for glucose sensing	PNIPAAm-*co*-N-(3-(dimethylamino)propyl) methacrylamide)	Amperometric	(51)
Ability to capture antibody and serve both as binding matrix and optical waveguide	PNIPAAm-*co*-MAA-BPMA terpolymer	Surface plasmon resonance	(52)
Thermoregulation of AuNP permeation that potentially increases the surface area available for functionalization with proteins	PNIPAAm-co_MBA	Optical detection by AVAC digital counting technology	This work

## 4 Conclusions

In the current study, we present a platform composed of a silicon wafer with a grafted layer of a thermosensitive PNIPAAm-*co*-MBA hydrogel. The hydrogel layer has been selected based on two characteristics that ensure the hydrogel’s compatibility with AVAC technology, namely, its permeability to AuNPs, and its optical properties that do not interfere with AuNP detection using AVAC technology. Regarding the permeability of the hydrogel to AuNPs, it has been demonstrated that the porosity of the hydrogel, adjustable through the cross-linker concentration, directly influences the thermoresponsive permeation behavior of the hydrogel toward AuNPs. In addition, three different deposition methods, drop casting, spin coating, and 3D printing, have been tested, and their influence on gel homogeneity has been evaluated, concluding that the drop casting method is the most effective method to achieve a uniform hydrogel coating. Furthermore, it has been shown that AuNPs embedded in the hydrogel layer can be correctly detected and characterized with the AVAC technology. This demonstrates that AVAC technology can be applied to hydrogel substrates and lays the groundwork for combining the three-dimensional structure and responsiveness of hydrogels with the precision of AVAC technology. The platform described in this work is a promising starting point for further development and optimization, and the next stages of research will involve the biofunctionalization of the hydrogel and the quantitative analysis of AuNP capture and biomarker detection.

## Supporting Information Available

Raman peaks located at 521 cm^–1^ of untreated and plasma-treated silicon substrate, FTIR spectra of PNIPAAm-*co*-MBA100 copolymers obtained at different reaction times, XPS spectra of PNIPAAm-*co*-MBA10 and PNIPAAm-*co*-MBA100 samples, O/C, N/C, and /O ratios and atomic concentration of C 1s, O 1s, and N 1s obtained by XPS high-resolution spectra for PNIPAAm-*co*-MBA10 and PNIPAAm-*co*-MBA100 samples, and WCA measurements carried out on PNIPAAm-*co*-MBA10 and PNIPAAm-*co*-MBA100 at *T* > LCST (PDF)
