# Correction to “Toward a Plasmon-Based Biosensor throughout a Thermoresponsive Hydrogel”

**DOI:** 10.1021/acsapm.5c01681

**Published:** 2025-06-12

**Authors:** Anne Parra, Óscar Ahumada, Andreas Thon, Valerio Pini, Julia Mingot, Elaine Armelin, Carlos Alemán, Sonia Lanzalaco

**Affiliations:** † 286054 Mecwins S.A., Ronda de Poniente, 15 2°D, Tres Cantos, 28760 Madrid, Spain; ‡ Department of Chemical Engineering, Universidad Politécnica de Madrid, 28006 Madrid, Spain; § IMEM-BRT’s Group, Departament d’Enginyeria Química, EEBE, 16767 Universitat Politècnica de Catalunya, C/Eduard Maristany, 10-14, Ed. I, 2nd floor, 08019 Barcelona, Spain; ∥ Barcelona Research Center in Multiscale Science and Engineering, EEBE, 16767 Universitat Politècnica de Catalunya, C/Eduard Maristany, 10-14, basement S-1, 08019 Barcelona, Spain; ⊥ Institute for Bioengineering of Catalonia (IBEC), The Barcelona Institute of Science and Technology, C/Baldiri Reixac 10-12, 08028 Barcelona, Spain

In our original article, one of the affiliations for the first author Anne Parra was missing. The affiliation has been corrected to Mecwins S.A., 28760 Madrid, Spain; Department of Chemical Engineering, Universidad Politécnica de Madrid, 28006 Madrid, Spain.

Please note that all other author affiliations and the current addresses remain unchanged from the original article. We would like to emphasize that this addition does not impact the conclusions, results, or interpretations presented in the original paper.

