# Supplementary material for: Toward a Plasmon-Based Biosensor throughout a Thermoresponsive Hydrogel
Source: ACS Appl Polym Mater. 2024 Nov 1;6(22):13618–29. doi: 10.1021/acsapm.4c02255 (PMC11590450; doi:10.1021/acsapm.4c02255)
Supplement: Supplementary file 1 — ap4c02255_si_001.pdf [file ap4c02255_si_001.pdf]

Supporting Information

Towards a plasmon-based biosensor throughout a thermo-responsive hydrogel

Anne Parra,[a] Óscar Ahumada,[a] Andreas Thon,[a] Valerio Pini,[a] Julia Mingot,[b,c] Elaine Armelin,[b,c] Carlos Alemán[b,c,d] and Sonia Lanzalaco[b,c]*

[a)] Mrs. Anne Parra, Dr. Óscar Ahumada, Dr. Andreas Thon, Dr. Valerio Pini,
Mecwins S.A.
Ronda de Poniente, 15 2°D, Tres Cantos
28760, Madrid, Spain

[b)] Dr. Sonia Lanzalaco, Prof. Carlos Alemán, Prof. Elaine Armelin, Mrs. Julia Mingot
IMEM-BRT's Group, Departament d'Enginyeria Química, EEBE,
Universitat Politècnica de Catalunya
C/ Eduard Maristany, 10-14, Ed. I, 2nd floor
08019, Barcelona, Spain.

[c)] Dr. Sonia Lanzalaco, Prof. Carlos Alemán, Prof. Elaine Armelin, Mrs. Julia Mingot
Barcelona Research Center in Multiscale Science and Engineering, EEBE,
Universitat Politècnica de Catalunya
C/ Eduard Maristany, 10-14, basement S-1
08019, Barcelona, Spain.

[c)] Prof. Carlos Alemán
Institute for Bioengineering of Catalonia (IBEC)
The Barcelona Institute of Science and Technology
C/ Baldiri Reixac 10-12
08028 – Barcelona (Spain)

*Corresponding authors: sonia.lanzalaco@upc.edu

Keywords: plasmonic detection, thermo-responsive hydrogel, gold nanoparticles, biomarker classification

MATERIALS

RESULTS AND DISCUSSION

Figure S1 shows the increase intensity of the Raman peaks located at ~521 cm^{-1}, corresponding to the second harmonic vibration of the absorption band located at ~950 cm^{-1}, discussed in the main text and attributed to the silicon substrate.

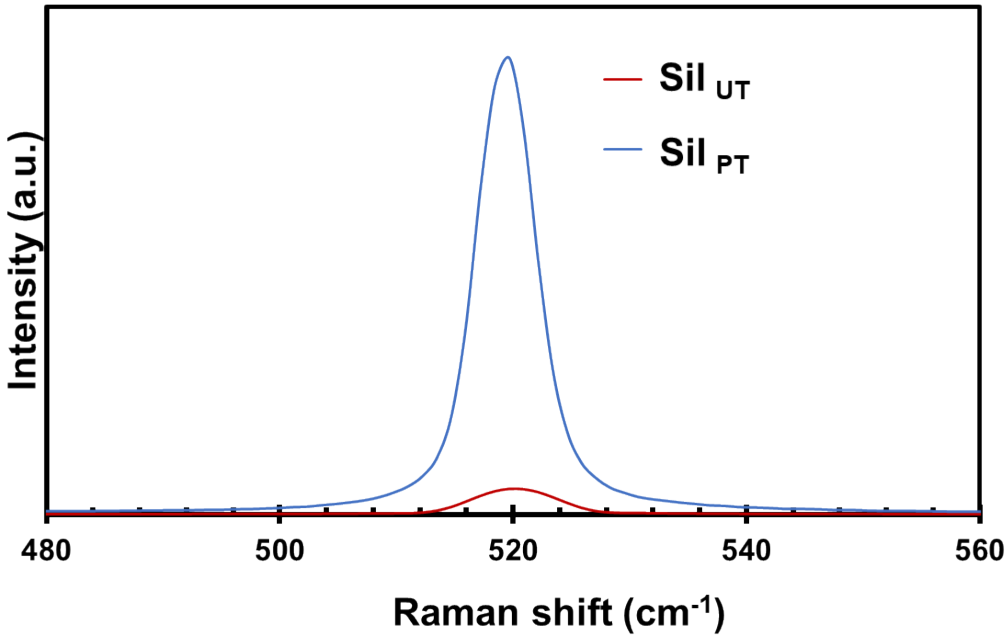

Figure S1. Raman spectra of untreated (UT) and plasma treated (PT) Sil wafers.

Figure S2 reports the FTIR spectra of PNIPAAm-co-MBA**100** copolymers obtained at different rection times (15, 30 and 60 min) showing a slight increasing of absorption bands corresponding to the thermosensitive hydrogel.

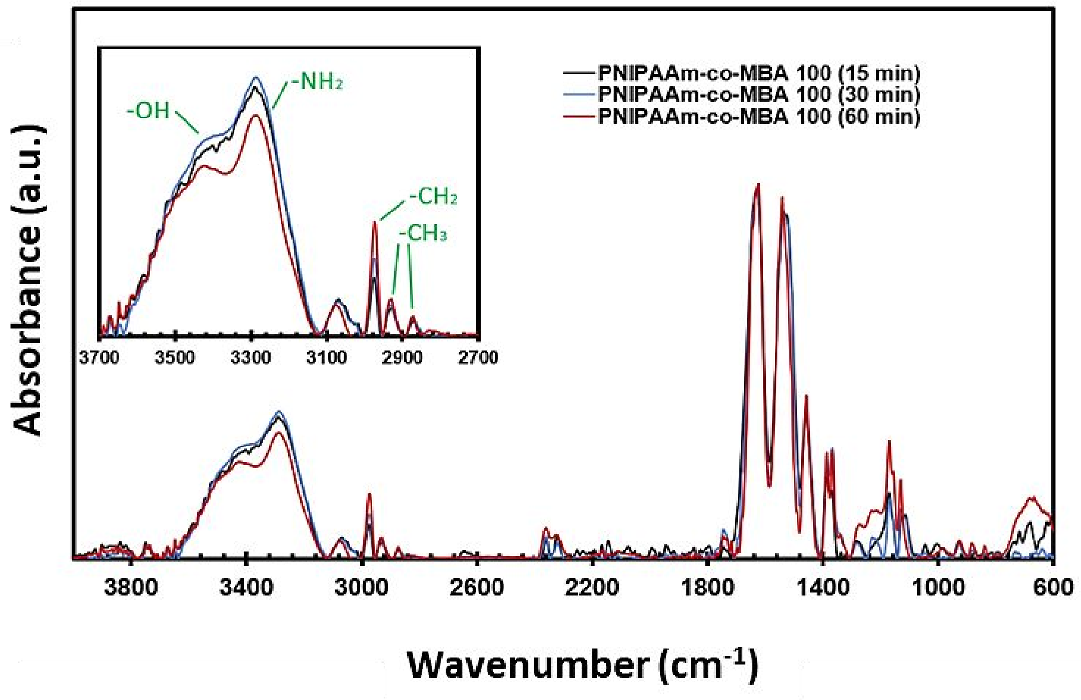

Figure S2. FTIR spectra of PNIPAAm-co-MBA 100 samples at different polymerization times (15, 30 and 60 minutes).

Figure S3 reports the wide-scan XPS spectra of (Sil)-g-(PNIPAAm-co-MBA**10**) and (Sil)-g-(PNIPAAm-co-MBA**100**) samples ay different crosslinker concentration. Three types of C species, the peaks at 285.0, 286.5 and 287.8 eV corresponded to carbons in C–C and C–H, C-N and C=O, respectively [1] of PNIPAAm-co-MBA. No peaks of the wafer were observed due to the uniform distribution of the gel and the surface sensitivity of the technique employed. The intensity of the peak centred at 287.8 eV slighltyl increases with the crosslinker concentration, due to the higher amount of MBA.

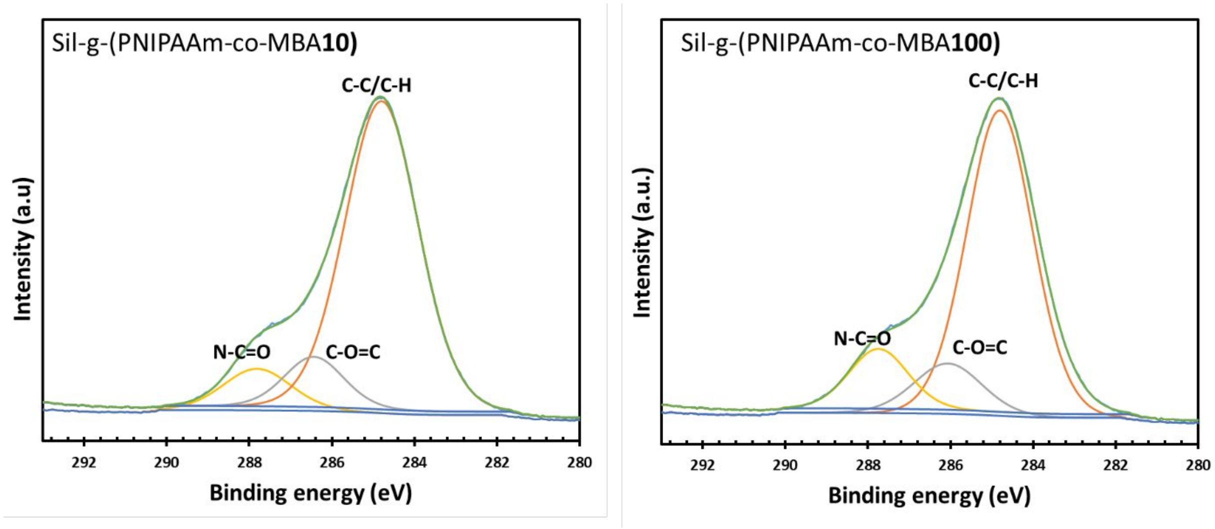

Figure S3. XPS spectra of PNIPAAm-co-MBA**10** and PNIPAAm-co-MBA**100** samples.

The atomic concentration of each chemical component was calculated and results are displayed in Table S1. Comparing to the theoretical composition values of NIPAM copolymerized with PNIPAAm as reported by literature [1], the element composition (atomic percentage, %) of carbon, nitrogen and oxygen are consistent. Both O/C and N/C ratios increase from Sil-(PNIPAAm-co-MBA**10)** to Sil-g-(PNIPAAm-co-MBA**100)**, confirming that higher amount of crosslinker is present in the final copolymers.

Table S1. O/C, N/C, N/O ratios and atomic concentration of C1s, O 1s and N 1s obtained by XPS high resolution spectra for PNIPAAm-co-MBA**10** and PNIPAAm-co-MBA**100** samples.

Sample code	Element	Atomic conc. (%)	O\C ratio	N\C ratio	N\O ratio
	C 1s	78.38			
PNIPAAm-co-MBA**10**	O 1s	11.31	0.14	0.13	0.91
	N 1s	10.31			
	C 1s	72.49			
PNIPAAm-co-MBA**100**	O 1s	13.52	0.18	0.19	1.04
	N 1s	13.99			

Figure S4 reports the water contact angle measurements carried out onto PNIPAAm hydrogel crosslinked with different amount of crosslinker and at T > LCST.

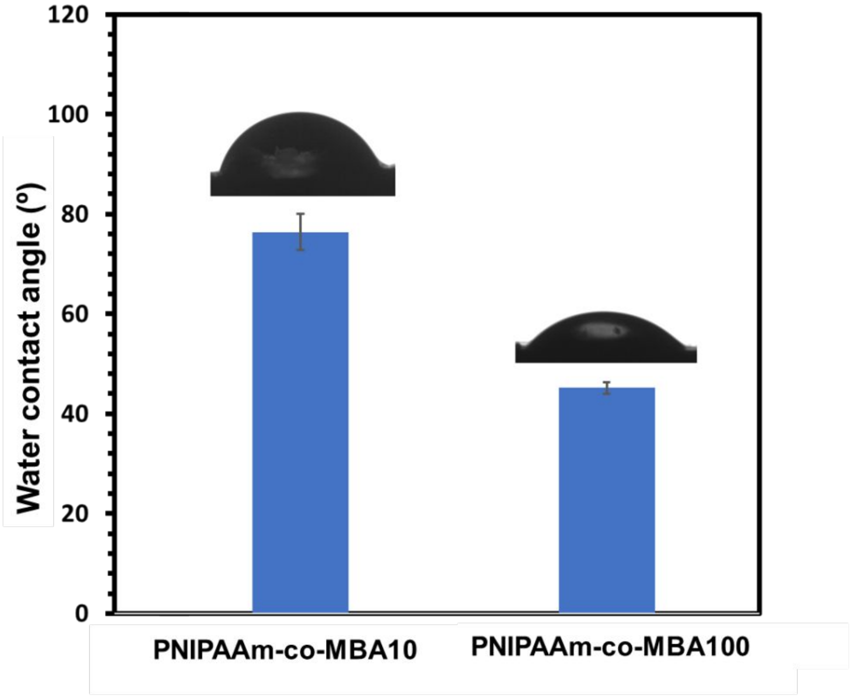

Figure S4. Water contact angles (WCA) measurements carried out on PNIPAAm-co-MBA10 and PNIPAAm-co-MBA100 at T > LCST.

REFERENCES

[1] X. Yang, Z. Sun, J. Gao, C. Yang, D. Tang, *Polym. Bull.* **2020**, *77*, 963.
